# Colorectal Cancer: Current and Future Therapeutic Approaches and Related Technologies Addressing Multidrug Strategies Against Multiple Level Resistance Mechanisms

**DOI:** 10.3390/ijms26031313

**Published:** 2025-02-04

**Authors:** Marianna Puzzo, Marzia De Santo, Catia Morelli, Antonella Leggio, Stefania Catalano, Luigi Pasqua

**Affiliations:** 1 Laboratory of Clinical, Biomolecular and Genetic Analyses Unit, Annunziata Hospital, 87100 Cosenza, Italy; marianna.puzzo@aocs.it (M.P.); stefania.catalano@unical.it (S.C.); 2 Department of Pharmacy, Health and Nutritional Sciences University of Calabria, Via P. Bucci, 87036 Arcavacata di Rende, Italy; marzia.desanto@unical.it (M.D.S.); catia.morelli@unical.it (C.M.); antonella.leggio@unical.it (A.L.); 3 NanoSiliCal Devices s.r.l., University of Calabria, 87036 Arcavacata di Rende, Italy; 4 Department of Environmental Engineering, University of Calabria, Via P. Bucci, 87036 Arcavacata di Rende, Italy

**Keywords:** colorectal cancer, molecular targeted therapy, multiple level resistance mechanisms, second level mutations directed targeted therapy, epidermal growth factor receptor

## Abstract

Colorectal cancer (CRC) is the third most common cancer and is associated with a poor prognosis. The mutation profile and related involved pathways of CRC have been, in broad terms, analyzed. The main current therapeutic approaches have been comprehensively reviewed here, and future possible therapeu-tic options and related technologies have been perspectively presented. The complex scenario represented by the multiple-level resistance mechanism in the epidermal growth factor receptor (EGFR) pathway, including mutations in KRAS, NRAS, and BRAF V600E, is discussed. Examples of engineered therapeutic approaches from the literature along with a drug combination tested in clinical trials are discussed. The encouraging results observed with the latter combination (the BEACON clinical trial), totally free from chemotherapy, prompted the authors to imagine a future possible nanotechnology-assisted therapeutic approach for bypassing multiple-level resistance mechanisms, hopefully allowing, in principle, a complete biological cancer remission.

## 1. Introduction

Colorectal cancer (CRC), a malignant tumor that forms in the tissue of the colon, is classified as the merging of colon cancer and rectal cancer because they present several common features. Colorectal cancer accounted for 12.7% of new cancer diagnoses and 12.4% of all cancer-related deaths across the 27 European countries in 2020 [1]. The estimated distribution in the EU of new cases of colorectal cancer is strictly dependent on the patients’ age group, and the survival rates for cancers diagnosed in 2018 has shown severe country-dependent heterogeneity [2]. 

Before 2000, bolus combinations of 5-FU/leucovorin were the North American standard of care for metastatic CRC. Successively, several randomized trials have successfully integrated oxaliplatin and irinotecan into previously existing 5-FU-based regimens for advanced CRC. The 5-FU and irinotecan combination (FOLFIRI) has shown an improved response rate and median overall survival, while the 5-FU and oxaliplatin combination (FOLFOX) exhibited an improved median progression-free survival and response rate, but not overall survival, when compared to 5-FU alone. In selected patients with a symptomatic tumor burden or those requiring tumor reduction to be surgical candidates, a feasible treatment option has been a combination of all three agents (FOLFOXIRI). This regimen has led to improved response rates and survival, although it is associated with increased toxicity, including neurotoxicity and neutropenia [3]. 

More recently, several studies have highlighted the importance of combining 5-fluorouracil (5-FU) with antioxidant molecules such as carotenoids, which have been shown to possess both protective antitumor effects [4] and promise as an approach to enhance the efficacy of chemotherapy in CRC, particularly in the context of drug resistance, including resistance to 5-FU. β-carotene, a carotenoid with antioxidant properties and potential antitumor effects, has been demonstrated to sensitize cancer cells to 5-FU by inhibiting the expression of specific ABC transporters, including ABCB1, ABCC9, and ABCC11. This inhibition leads to an increase in the intracellular concentration of 5-FU, thereby enhancing its therapeutic efficacy. Moreover, β-carotene can induce cell cycle arrest at the G2/M phase and promote apoptosis in cancer cells, further contributing to its therapeutic action [5].

At the beginning of the century, the first targeted agents for CRC were approved by the Food and Drug Administration (FDA). In 2004 cetuximab was approved, followed by panitumumab in 2006, both as anti-EGFR agents. In the same year, bevacizumab was also approved, and later, between 2012 and 2015, ziv-aflibercept, regorafenib and ramucirumab were introduced as anti VEGFR agents [6]. 

Even as the ideal combinations and sequences of these regimens were elucidated, targeted therapies such as bevacizumab cetuximab and panitumumab were added to treatment protocols, leading to favorable outcomes [7]. In general, most patients (70–80%) newly diagnosed with CRC have localized disease that is amenable to curative (R0) surgical resection [8]. After R0 resection, adjuvant chemotherapy with cytotoxic agents has been recommended as standard clinical practice for patients with stage III CRC [9]. This recommendation was supported by a pooled analysis of data from the National Surgical Adjuvant Breast and Bowel Project (NSABP) trials [10], which demonstrated significantly improved survival outcomes after a combination of surgery and chemotherapy compared with surgery alone (*p* < 0.0001). The remaining 20% to 30% of newly diagnosed patients presented with unresectable metastatic disease. Furthermore, a large proportion of patients (40–50%) experienced disease recurrence after surgical resection or develop metastatic disease, typically in the liver or lungs [11]. 

The management of patients with metastatic CRC (mCRC) requires the systemic administration of cytotoxic drugs [9]. Patients with unresectable mCRC who receive supportive care alone have been shown to have a poor prognosis, with a median overall survival (OS) of 5 months [12]. In contrast, patients with mCRC who receive chemotherapy have been shown to have a median OS of more than 2 years [13]. 

The problem of defining the prognosis of the different CRC subtypes has been approached through the Consensus Molecular Subtype (CMS) classification that considers tumor pathological characteristics and gene expression. Although it is still being defined, it can drive drug development and application [14,15,16]. In the meantime, the drug engineering advancements allow the presentation of antibody drug conjugates (ADCs) able to produce synergistic antitumor activity between HER2 antibodies and chemotherapy for treating advanced colorectal cancer [17]. 

In this feature paper, a possible nanotechnology-assisted route of development through mesoporous silica of a multi-drug targeted therapy is hypothesized.

Mesoporous silicas are amorphous silica nanohoneycombs with regular porosity whose diameter size is plannable in the mesoporous range (2–50 nm). They have been extensively studied for their high specific surface and pore volume distribution all over the world since their introduction in the 90s. These features make possible several kinds of applications as molecular sieves; they are, in fact, able to capture biological molecules from body fluids [18,19], act as substrates for developing stimuli-responsive materials [20], and provide a starting architecture for bionanotechnology [21]. 

The materials engineering approach deals with the emerging ability of creating matter at the nanoscale, obtaining new materials that are themselves devices. In the bionanotechnology field, for instance, an accurate interdisciplinary evaluation of the biological conditions is also requested [22].

We discuss the elements that could define the hopeful scenario of a possible enfranchisement of CRC treatments from chemotherapy, glimpsing a complete recovery with fully biological instruments by means of a multidrug inhibiting the initiation, progression, and migration of CRC. The hypothesized therapy would benefit from drug synergies, helping to prevent resistance and thereby expanding its potential for treating CRC. In principle, it represents a completely biological treatment for CRC, with the possibility of being translated to other malignancies. 

## 2. Causes of CRC

The majority of CRC cases are not inherited (familial versus hereditary, about 30/10) but sporadic, whose cause is related to risk factors such as increasing age, smoking, excessive alcohol consumption, obesity, a diet rich in fats, precancerous colon polyps (adenomas) and other undefined risk factors [23].

**Figure 1:**
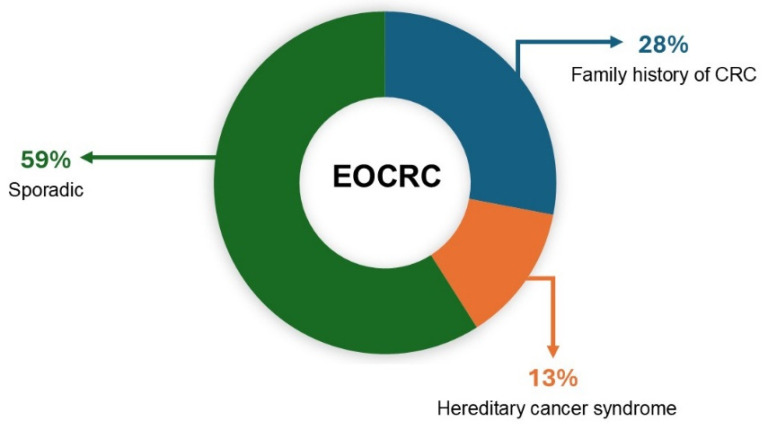
Proportions of early-onset colorectal cancer (EOCR) associated with sporadic and hereditary factors.

Early-onset colorectal cancer (EOCRC), defined as cases diagnosed before the age of 50, accounts for 10–12% of all new colorectal cancer (CRC) diagnoses and shows an increasing incidence not related to any known cause. Pathogenic germline variants in known cancer predisposition genes are present in ≈13% (range: 9–26%) of EOCRC, while 2.5% of patients exhibit germline pathogenic variants in hereditary cancer genes not usually associated with a CRC predisposition. On the other side, 28% of EOCRC patients have a family history of the disease. The evidence gathered supports the recommendation that all patients diagnosed with an EOCRC should be referred to a specialized genetic counseling service and offered somatic and germline pancancer multigene panel testing. The identification of a germline pathogenic variant in a known hereditary cancer gene has relevant implications for the clinical management of the patient and his/her relatives, and it may guide surgical and therapeutic decisions [9]. In Figure 1, the inherited and familial components of early-onset colorectal cancer are presented. 

## 3. CRC: From Pathogenesis to Molecular Typing

Approximately one-third of colorectal cancer cases in the United States are linked to a family history of the disease. The presence of adenomatous polyps in the colon is a significant risk factor for CRC. Polyps removal during colonoscopy reduces the risk of progression to cancer [9]. First-degree relatives of patients with colorectal adenomas or invasive colorectal cancer are at an increased risk of developing the disease. Additionally, genetic syndromes such as Lynch syndrome (Hereditary Nonpolyposis Colorectal Cancer, HNPCC) and Familial Adenomatous Polyposis (FAP) are strongly associated with CRC [9]. 

Lynch Syndrome arises from mutations in DNA mismatch repair genes (MMR) like MLH1, MSH2, MSH6, and PMS2 that can lead to an increased incidence of CRC and other cancers. The other genetic condition, FAP, is caused by mutations in the APC gene, resulting in the formation of numerous polyps in the colon, many of which have the potential to become cancerous [23]. Additionally, a family history of colorectal cancer can elevate the risk, even though this form is not hereditary [9]. Genetic and environmental factors are intricately intertwined in CRC development, as genetic predispositions and environmental exposures shape host–microbiota interactions, contributing to intestinal immune dysfunction [24].

Sporadic colorectal cancer (sCRC) represents the most prevalent form of this disease and poses a substantial burden to public health and healthcare systems. Sporadic CRC accounts for approximately 59% of all colorectal cancer cases. Its incidence is rising in many populations, particularly among individuals over the age of 50. However, an increasing trend is also observed among younger adults [9].

CRC is influenced by several risk factors, including lifestyle and dietary habits. Diets high in fat and low in fiber, particularly those rich in red and processed meats and low in fruits and vegetables, have been linked to a higher risk of CRC. Furthermore, alcohol consumption, along with smoking, significantly increases the likelihood of developing CRC. Obesity and sedentary lifestyles are also notable risk factors, as obesity promotes inflammation and disrupts metabolic pathways, further contributing to CRC development [23].

Another risk factor for CRC is associated with inflammatory bowel disease (IBD), which differs in its causation and pathogenesis from sCRC. Chronic inflammatory diseases such as ulcerative colitis and Crohn’s disease elevate the CRC risk. Chronic inflammation generates oxidative stress and DNA damage, activating oncogenes and deactivating tumor-suppressor genes. The duration of the disease significantly increases the risk. Cumulative CRC risks are 2% at 10 years, 8% at 20 years, and 18% at 30 years for patients with ulcerative colitis. The extent of colonic involvement is correlated with higher neoplastic risk. Other important risk factors include a family history of CRC, primary sclerosing cholangitis, male gender, and a younger age at the time of IBD diagnosis [24].

Pollution and exposure to toxic chemicals, such as certain pesticides and industrial solvents, may increase the CRC risk. To conclude, some studies suggest that hormone replacement therapy (HRT) in women may influence CRC risk, although the findings remain inconsistent. 

All these risk factors underscore the critical importance of prevention, early detection, and regular screening, particularly for individuals with a family history of colorectal cancer or other significant risk factors, such as chronic intestinal inflammation [9].

The pathogenesis of CRC is a multifaceted process involving a series of molecular and cellular events that transform cells into malignant ones. On one hand, germline or somatic variants predispose to cancer. For example, mutations in the APC gene (and subsequent activation of the Wnt signaling cascade), like those discussed above, represent one of the earliest events in colorectal carcinogenesis, driving the formation of adenomatous polyps [25,26]. On the other hand, chronic inflammatory conditions, such as ulcerative colitis and Crohn’s disease, can similarly contribute to carcinogenesis through mechanisms of persistent inflammation, which induce DNA damage and promote cellular proliferation [27,28]. 

**Figure 2:**
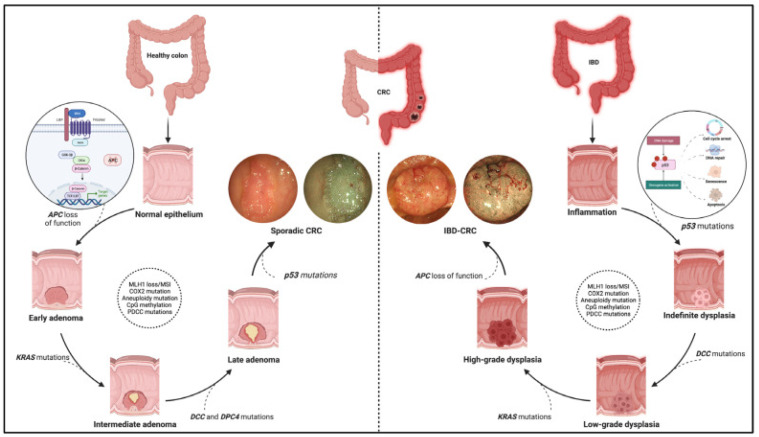
Different pathogenic pathways of sporadic and IBD-associated colorectal cancer. The main genetic mutations characterizing the advancement of the two different kinds of cancers are also indicated. Source: Nardone, O.M. et al. [24].

Furthermore, the progression of adenomatous polyps to invasive colorectal cancer is a gradual process characterized by the accumulation of genetic mutations and epigenetic alterations. This progression is often described by the "adenoma-carcinoma" model, in which adenomatous polyps may evolve into carcinoma in situ and subsequently into invasive carcinoma [24]. New possibilities for personalized therapies derive from the comprehension of the genetic and molecular mechanisms of CRC associated with inflammatory bowel disease (IBD) other than those from the advancement of the new OMIC techniques. For instance, the elevated expression of *Oncostatin M* (OSM), a group of pleiotropic cytokines, has been associated with a poor response to TNFα blockers in certain patients with IBD, suggesting its potential as both a biomarker and a therapeutic target [24]. The multi-omics approach, encompassing genomics, transcriptomics, proteomics, and metabolomics, provides opportunities to identify novel biological mechanisms, clinically relevant biomarkers, and integrated signatures for patient stratification. Figure 2 illustrates the different pathogenesis pathways for sporadic CRC and IBD-CRC, along with the associated mutations at each step in both the pathways. The adenoma to carcinoma sequence characterizes sporadic CRC, while IBD progresses through different degrees of dysplasia, leading to colitis-related CRC [24].

## 4. Dependence of Progression and Therapeutic Options on Tumor Microenvironment. The Four Consensus Molecular Subtypes

CRC molecular classifications are based on both genetic and epigenetic characteristics. Mutations, microsatellite instability (MSI), CpG island methylator phenotype (CIMP), chromosomal instability (CIN), copy-number deviations (SCNA) and the most significant pathways that affect CRC initiation and progression, such as WNT and MYC, are considered. 

The newly acquired potentialities in genomic, transcriptomic, and big-data technologies allow the investigation of the molecular characteristics of tumors. The main items concern CRC development, whose transition from benign to malignant lesions is based on the acquisition of a series of mutations over time, and is induced by some key driver genes. Among them, the adenomatous polyposis coli (APC) gene, accompanied by its mutations, regulates growth advantages in epithelial cells and results in the formation of a small adenoma. Successively, KRAS and BRAF mutations determine the cell’s expansion, which produces the transformation to a large adenoma. Finally, PIK3CA, SMAD4, and p53 mutations develop the malignancy, with the potential for invasiveness and metastasis [25].

Microsatellite instability (MSI) is another hallmark of CRC’s mutational landscape, arising from defects in DNA repair genes like MLH1, MSH2, MSH6, and PMS2. MSI-high (MSI-H) tumors tend to respond well to immune checkpoint inhibitors and exhibit distinct mutational profiles with a high mutation burden, often linked to Lynch syndrome [29,30,31,32,33]. Mutations in the MLH1 or MSH2 genes are associated with an increased risk of developing cancer, while mutations in the MSH6 or PMS2 genes have a comparatively lower risk of cancer development [34]. Nearly 15–20% of primary CRCs have the MSI phenotype, whereas the remainder are microsatellite stable (MSS). The presence of MSI-H, if compared to MSI-L or MSS status, in CRC is associated with a superior anti-tumor immune response, inhibition of tumor cell growth, and an improved prognosis, representing a predictor to be considered when selecting a treatment strategy for MSI-H and MSI-L [25]. 

Chromosomal structural variants, such as (CIN), are also prevalent in sporadic CRC cases. CIN is characterized by chromosomal number and structure abnormalities, leading to loss of heterozygosity (LOH) and copy number variations, which drive tumor progression [32]. CIN, nominated as the suppressor pathway phenotype, is observed in 70–85% of CRC tumors and is often considered equal to MSS status [35]. On the other hand, CIMP is an epigenetic event, described by Toyota et al. [36], that precedes the onset of cancer. Mutations in genes such as MLH1 and CDKN2A are common in this context and influence the therapeutic response [32]. They feature an increase in methylation levels within the promoter regions that can lead to the silencing of tumor suppressor genes. 

**Figure 3:**
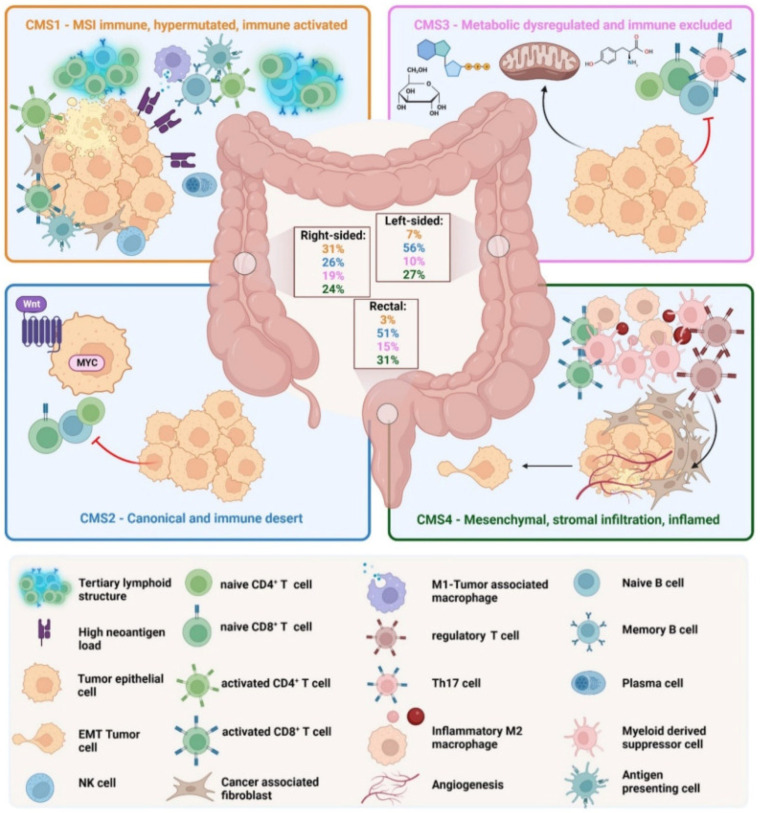
Graphical representation of the four different molecular subtypes. Source: Braumüller, H. et al. [37].

CRC has been classified according to four different molecular subtypes [14,37,38]. The different subtypes are represented in Figure 3. 

The MSI immune subtype, or MSI-like subtype, is characterized by infiltration of lymphocytes and the formation of tertiary lymphoid structures. It presents mutations in the MLH1 and BRAF genes. A diffuse immune infiltrate of T helper 1 (TH1) cells, natural killer (NK) cells and cytotoxic T lymphocytes (CTL) is present. 

CMS1 also shows a CIMP phenotype and presents an incidence, among total CRCs, of approximately 14%. CMS2, the canonical subtype, includes CRCs with higher CIN and a high level of SCNA. CMS2 CRCs show an upregulated WNT and MYC pathway and no dendritic cell (DC) recruitment. The CMS2 subtype is poorly immunogenic. There are no immune infiltrates and no immune regulatory cytokines. Approximately 37% of CRCs belong to CMS2. CMS3, a metabolic subtype, is characterized by immune cell exclusion and dysregulation of the glucose pentose, nitrogen, fatty acid and several other metabolic pathways. Compared with other CMS phenotypes, CMS has low CIN and CIMP status and higher KRAS mutations. Approximately 13% belong to the CMS3 subtype. CMS4, the mesenchymal subtype, is characterized by high CIN and a strong expression of the epithelial–mesenchymal transition (EMT). CMS4 tumors show TGF-β signaling and a high C-X-C chemokine ligand 12 (CXCL-12) expression. It has high levels of infiltrating CTLs, macrophages and stromal cells. Approximately 23% of CRC tumors fall into CMS4. Although there are high levels of leukocyte infiltration, patients with CMS4 tumors have the worst prognosis among the four subtypes. 

The integrative analysis of mutations and copy-number variations based on The Cancer Genome Atlas (TCGA) data showed that BRAF mutations are frequently found in CMS1 and are related to the MSI phenotype while KRAS mutations are frequently seen in CMS3. Furthermore, the receptor tyrosine kinase (RTK) and mitogen-activated protein kinase (MAPK) pathways are generally activated in CMS1 and CMS3, although none of these genetic aberrations exclusively belong to specific CMS subtypes.

A fluoropyrimidine-based cytotoxic chemotherapy combination in association with a biological and/or targeted agent is the current, first-line treatment in metastatic CRC (mCRC) depending on the individual molecular status.

The best prognosis belongs to CMS2, whereas CMS1 tumors present a higher risk of disease progression and death after chemotherapy. CMS1 responds to immunotherapy due to mesenchymal features and immunosuppressive molecules while CMS4 has the worst prognosis. 

A comprehensive analysis, with an increasing understanding of CRC genomic complexity, already in course in the last few years, incorporating the definition of the main drivers involved in intrinsic and acquired resistance to therapies may hopefully contribute to reaching an awareness for switching from “one marker–one drug” to “multi-marker drug combinations”, allowing oncologists to give “the right drug to the right patient” [25,39]. Wider biomarker-driven clinical trials considering the “dynamic clonality of CRC” [39] can represent the ultimate instrument for this transition. 

In summary, although most of the available CMS data are based on retrospective trials, CMS shows higher heterogeneity than the standard genomic information, defining a path to the maximization of the personalized therapies.

## 5. CRC: Critical Pathways and Mutation Landscape

In the molecular profiling context of colorectal cancer (CRC), multiple oncogenic pathways and mutational mechanisms have been identified as critical for disease pathogenesis, progression [29,31,33,40,41], treatment response, and chemoresistance, often serving as prognostic and sometimes predictive markers for therapy.

The mutational profile of CRC, as observed by Alex J. Cornish et al. [42], is characterized by a variety of somatic mutations that influence disease progression and the treatment response. The mutational landscape of CRC is highly heterogeneous, meaning that different tumors can exhibit distinct mutational profiles even within the same population. This heterogeneity affects treatment outcomes and prognosis [31].

Among the detected mutations, several directly involve genes implicated in key pathways. These mutations allow the identification of patient subsets with varying therapeutic responsiveness. For example, mutations in the APC (adenomatous polyposis coli) gene, crucial for the adenoma–carcinoma transition (Figure 2) [33,40], lead to aberrant activation of β-catenin, driving cellular proliferation [29,30,31,32,33]. Similarly, SMAD4 mutations, common in CRC, are pivotal for neoplastic transformation and therapeutic response [33]. Mutations in the TP53 gene, encoding the tumor suppressor p53, are frequently observed in CRC. Loss of p53 function allows tumor progression, with TP53 mutations linked to more aggressive tumor behavior [3,30,31,43].

Among the various molecular mechanisms of chemotherapy resistance in colorectal cancer, the dysregulation of key signaling pathways, such as EGFR, PI3K, and mTOR, plays a prominent role. These pathways can undergo modifications that facilitate the evasion of chemotherapeutic responses. Specifically, mutations in critical genes, including KRAS and PIK3CA, can alter therapeutic sensitivity and contribute to resistance [44]. Additional mechanisms, such as the overexpression of ATP-binding cassette (ABC) transporters, can reduce the intracellular concentration of cytotoxic drugs, thereby diminishing their efficacy [44,45]. Moreover, the tumor microenvironment (TME) significantly influences the chemotherapy response by creating conditions conducive to treatment resistance. On one hand, M2-type macrophages contribute to tumor growth and induce an immunosuppressive environment, which can reduce the efficacy of therapies, including chemotherapy. On the other hand, exosomes derived from neoplastic cells can play a role by dispersing chemotherapeutic agents away from the tumor site [46]. Moreover, compensatory feedback loops further exacerbate this issue, as therapeutic failure often activates alternative pathways that increase resistance. These mechanisms collectively emphasize the complexity of drug resistance and highlight the need for combinatorial therapeutic strategies to effectively overcome resistance in CRC treatment [45,46].

### 5.1. The EGFR Pathway

The EGFR (epidermal growth factor receptor) pathway in colorectal cancer (CRC) is a crucial signaling cascade that plays a central role in tumor growth and progression. It is fundamental to various cellular functions, including proliferation, survival, invasion, and immune response. Aberrant activation of EGFR signaling is frequently associated with pathological conditions such as colorectal carcinoma, where it contributes significantly to tumor development and metastasis [29,30,31,32,33,34,35,36,37,38,39,40,41,42,43,47,48,49,50,51].

The pathway begins with the binding of epidermal growth factor (EGF) to the extracellular domain of EGFR, a receptor belonging to the human epidermal growth factor receptor (HER) family, also referred to as HER1 [52]. This ligand–receptor interaction induces receptor dimerization and activates its intrinsic kinase activity [47]. Upon activation, EGFR recruits various adaptor proteins, such as GRB2, which trigger downstream signaling pathways, including the MAPK (mitogen-activated protein kinase) and PI3K/AKT pathways [35,48].

The RAS–RAF–MAPK cascade plays a key role in regulating cell proliferation and differentiation. Within this cascade, RAS activation initiates a sequence of events involving the activation of RAF, MEK, and ERK, culminating in the regulation of gene transcription. Concurrently, the PI3K–AKT pathway is activated through PI3K, leading to the production of PIP3 and subsequent activation of AKT, a major regulator of cellular growth and survival that inhibits apoptosis [48].

Another signaling branch involves the activation of PLC-gamma. When EGFR is activated, PLC-gamma facilitates the production of inositol trisphosphate (IP3) and diacylglycerol (DAG), critical mediators of calcium signaling and kinase activation [49].

Additionally, EGFR activation can modulate other pathways, such as SRC and STATs, which are essential for processes like angiogenesis, invasion, and metastasis [51].

Biological agents, such as the anti-EGFR monoclonal antibodies cetuximab/panitumumab and the anti-VEGF monoclonal antibodies bevacizumab (BV) and ramucirumab, are added to cytotoxic drugs. Cetuximab is a chimeric immunoglobulin G (IgG) antibody inducing EGFR internalization and degradation once bound to the external domain of EGFR [52]. The fully humanized antibody panitumumab, differently from murine–human chimeric antibodies, like cetuximab, that might cause immunogenic reactions, does not trigger antibody-dependent cell-mediated cytotoxicity [53].

Likewise, mutations in the KRAS gene are present in over 30% of CRC patients [43], particularly affecting codon 12. Common variants like G12C and G13D are associated with a poor prognosis and reduced responsiveness to EGFR inhibitors. These mutations impact the MAPK signaling pathway [29,30]. However, therapies such as sotorasib, targeting the KRAS p.G12C mutation, have expanded treatment options for patients previously unresponsive to biological therapies.

Mutations in PIK3CA and other alterations in the PI3K/AKT pathway contribute to tumor growth and treatment resistance [29,31,32,43]. Interestingly, a mutual exclusivity analysis across populations revealed that PIK3CA and KRAS mutations co-occur more frequently in Caucasians, while APC and TP53 mutations are prevalent in the Taiwanese cohort. This suggests alternative carcinogenic pathways and genetic drivers across populations [40].

Mutations in BRAF, particularly the V600E variant, occur in 5–12% of CRC cases. These mutations are associated with a poor prognosis and resistance to anti-EGFR therapies, particularly in microsatellite-stable (MSS) tumors [32,33,41]. Additionally, studies such as that by Ebtehal Alsolme et al., also highlight somatic mutations in DNA repair and signaling pathway genes, including BRCA2, CHEK1, ATM, ATR, and MYCL [30].

### 5.2. The VEGFR Pathway

The VEGF family is a key regulator of tumor angiogenesis. It is composed of five growth factors: VEGF-A, VEGF-B, VEGF-C, VEGF-D, and placental growth factor (PlGF). These growth factors differentially bind and activate three cell surface tyrosine kinase receptors, VEGFR-1, VEGFR-2, and VEGFR-3 [54,55]. 

VEGFR-1 is a member of the receptor tyrosine kinase family expressed on many kinds of cells, including epithelial cells, inflammatory cells, and cancer cells. It shows high affinity for VEGF-1 and relatively low affinity for VEGF-2, and PIGF seems to contribute mainly to cell differentiation and migration rather than cell proliferation during vascular formation [56].

Contrarily, VEGFR-2, actively involved in vascular formation, is mostly expressed on blood and lymphatic epithelial cells. VEGFR-2 mainly interacts with VEGF-A, and after activation, VEGFR-2 leads to tyrosine residue phosphorylation and activation of several pathways, such as the PLCγ, RAS/RAF/ERK/MAPK, and the PI3K/AKT pathways, by which cell apoptosis may be avoided [57,58].

VEGFR-3, activated by VEGF-C and VEGF-D, contributes relatively independently to lymphatic vessel formation. If activated, it mediates the differentiation, migration, proliferation and survival of lymphatic endothelial cells by activating the RAS/MAPK/ERK pathway and the PI3K–AKT/PKB pathway [59,60,61].

A lack of patient response to anti-VEGF therapies, tumor regrowth and disease progression indicate the evasion of therapeutic inhibition of angiogenesis by cancer cells due to the possible development of resistance by one or more alternative angiogenic contributing pathways. Recently "agnostic" antibodies developed using histology-independent models, such as pembrolizumab and nivolumab—two programmed death receptor-1 (PD-1) blocking antibodies—along with larotrectinib and entrectinib, which are indicated for solid tumors with neurotrophic receptor tyrosine kinase (NTRK) gene fusions, have also been approved for the treatment of mCRC.

Bevacizumab, a humanized IgG monoclonal antibody introduced in 2004, was the first anti-VEGF/VEGFR agent. The emerging agents include aflibercept, ramucirumab and regorafenib [6]. 

Aflibercept is a VEGFR-1 and VEGFR-2 extracellular domain recombinant fusion protein that acts as a ligand trap targeting VEGF-A, VEGF-B, and PIGF. It shows a stronger affinity for VEGF-A than bevacizumab [62], and the details of its action mechanism will be discussed below in Section 6.2. 

Ramucirumab is a fully humanized monoclonal VEGFR-2-targeted IgG antibody used in combination with FOLFIRI [63]. Regorafenib is a multiple-target TKI approved by the FDA to treat metastatic CRC. It acts on VEGFR, PDGFR (platelet-derived growth factor receptor), FGFR (fibroblast growth factor receptor), and BRAF [6].

Anti-VEGF resistance is observed in several kinds of cancer, including CRC. It is due to the activation of alternative signaling pathways and the excretion of different angiogenesis-related proteins. PIGF is a crucial factor in overcoming anti-VEGF resistance due to its upregulation and overexpression in CRC resistant to antiangiogenic therapies. This role is also confirmed by the better performance of aflibercept when compared to bevacizumab in xenograft models [64]. VEGF-targeted therapies for resistant cancers, including CRC associated with resistance to bevacizumab, show an increasingly controlled proliferation and progression in preclinical studies upon targeting of both VEGF and angiopoietin-2; in fact, the angiopoietin/TIE (tyrosine kinase with Ig-like and EGF-like domains) signaling RTK pathway, involved in vascular formation and stabilization by mediating the downstream RAS/RAF and PI3K/AKT pathways, is negatively regulated by angiopoietin-2 [65,66,67,68].

### 5.3. The HGF/C-MET Pathway

Hepatocyte growth factor (HGF), whose tissue and serum expression levels are related to a poor prognosis of CRC patients, is secreted mostly from mesenchymal tissues and is currently the only known ligand for the receptor tyrosine kinase known as mesenchymal–epithelial transition factor (c-MET or MET) that is active in tumor proliferation, survival, metastasis, and acquired drug resistance [69]. 

The activation of the HGF/MET pathway, which starts from HGF binding to the MET receptor on the membrane, initiates various downstream signal transduction pathways, including the MAPK/ERK, PI3K/AKT, and STAT/JAK pathways and the nuclear factor-κB complex, to regulate hematopoiesis, organ regeneration, and wound healing [70,71,72,73]. Another major method of regulating signaling activity relies on crosstalk between the MET pathway and other RTKs, especially EGFR. Overexpression of both MET and EGFR is commonly found in the same malignant tumor, such as CRC [74]. 

MET was the first factor to be identified as responsible for EGFR inhibitor resistance, even in the absence of known resistance related mutations [75,76,77,78]. The HGF/c-MET pathway is a promising site for targeted therapy. Its blockage can occur via monoclonal antibodies or small molecules according to different pharmacological mechanisms. Drugs can either block HGF activation and production or interfere with the binding of HGF to MET receptors, both competitively binding to MET receptors (MET antagonists) and inhibiting intracellular tyrosine kinase activity (MET TKIs); in the latter case, several drugs (small molecules) functioning as selective or nonselective TKIs can be adopted thanks to their similar RTK structure to MET that guarantee their pharmacological effects.

## 6. Advanced Therapeutic Approaches

In this section, different advanced approaches to CRC therapeutics and related action mechanisms are presented.

### 6.1. Antibody–Drug Conjugates 

Antibody–drug conjugate (ADC) is an engineered kind of drug that contains an antigen-specific antibody and a cytotoxic payload, which can provide an improved performance in terms of the survival time of tumor patients [79]. To date, there are several HER2-ADC products on the market, for which two anti-HER2 ADC (trastuzumab emtansine and trastuzumab deruxtecan) have been authorized by the FDA for distinct types of HER2-positive carcinoma in the breast [80].

**Figure 4:**
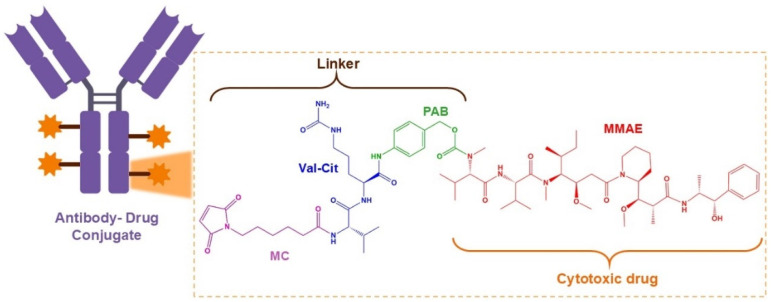
Molecular structure of disitimab vedotin (RC48) involving a derivate antibody–drug conjugate comprising the antibody (disitamab) and cytotoxic drug (monomethyl auristatin E, MMAE) linked by a MC-Val-Cit-PAB cleavable linker. It contains a thio reactive maleimidocaproyl (MC) group, a protease-sensitive valine-citrulline (Val-Cit) dipeptide, and a para-aminobenzyl carbamate (PAB) spacer.

**Figure 5:**
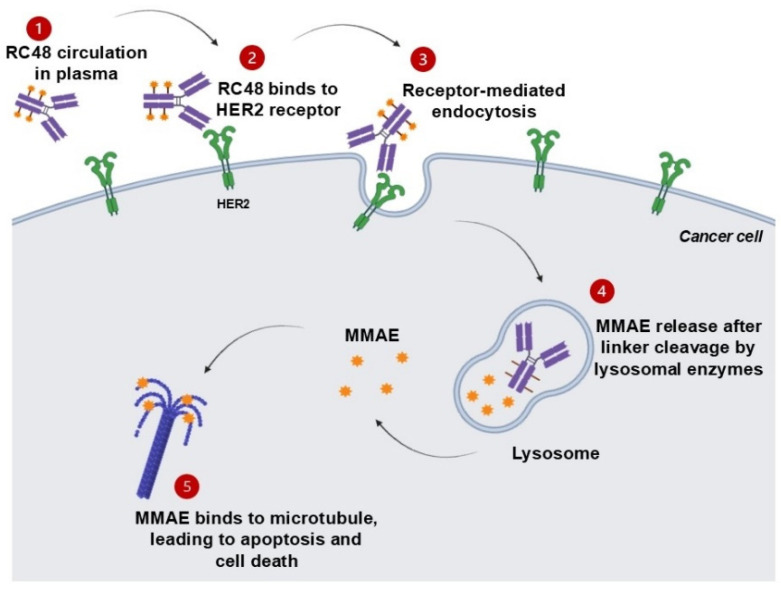
The anticancer mechanism of RC48. Upon administration, RC48 circulates in the bloodstream (1) and exhibits a high affinity toward the HER2 receptor overexpressed on tumor cells, forming the RC48–HER2 complex (2). This complex is then internalized through receptor-mediated endocytosis (3). Within lysosomes, the cytotoxic payload (MMAE) is released when the linker is cleaved by the cell’s intracellular conditions (4). The released MMAE disrupts microtubules, leading to apoptosis (5).

Disitamab vedotin (RC48) is a newly developed ADC drug targeting HER2 that is comprised of hertuzumab coupling monomethyl auristatin E (MMAE) via a cleavable linker. Either alone or in combination with gemcitabine (GEM) in various models of HER2-positive advanced CRC, it showed synergetic antitumor activity in vitro and in vivo [17]. The molecular structure and anticancer mechanism of action of RC48 are depicted in Figure 4 and Figure 5, respectively.

### 6.2. Aflibercept

**Figure 6:**
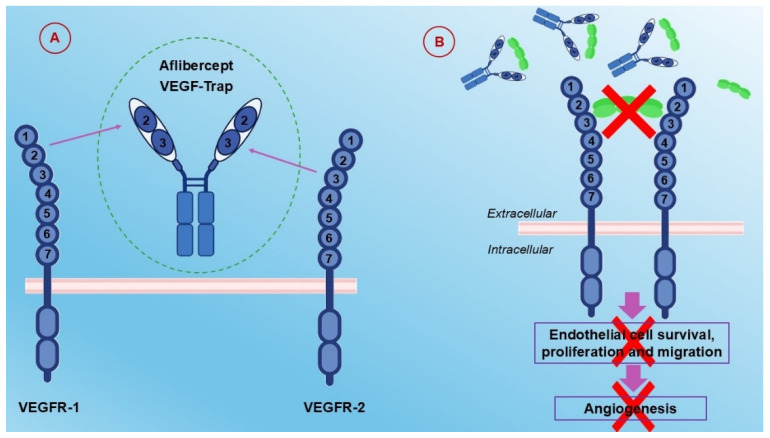
Structure and Mechanism of Action of Aflibercept. (**A**) Aflibercept is a fusion protein comprising domain 2 of VEGFR-1 and domain 3 of VEGFR-2, linked to the Fc region of IgG1. (**B**) Aflibercept works by binding to VEGF and preventing VEGF’s interaction with its native receptors, VEGFR1 and VEGFR2. As a result, endothelial cell migration and proliferation are inhibited, effectively halting angiogenesis.

Aflibercept is a multiple angiogenic factor trap designed to block the angiogenesis network. It is a recombinant fusion protein, working as an angiogenic factor trap that blocks the binding of VEGF-A, VEGF-B, and placental growth factor (PlGF) [81,82,83]. It has been approved for use in combination with FOLFIRI in the treatment of mCRC that is resistant to or has progressed after an oxaliplatin-containing regimen [84]. The structure and mechanism of action of aflibercept are presented in Figure 6.

## 7. Opportunities for CRC Through Nanotechnology: Pharmacological Needs and Possible Synergetic Actions to Overcome Multiple Level Resistance Mechanisms

### 7.1. Nanotechnology Solutions for CRC

Nanomedicine, the application of nanotechnology to achieve innovation mostly in therapeutics and diagnostics, where it offers solutions with improved performance, exploits the abilities of nanostructured materials to interact with biological structures by crossing natural barriers and interfering, in a planned way, with biological mechanisms. The most common form of device able to manage the release of a drug is usually a nanoparticle. Several types of nanoparticles can be developed, and the corresponding starting nanoarchitectures can vary significantly among them [85]. With regard to CRC, several different solutions will be not-comprehensively described [86]. Drug encapsulation has been tested in nanodevices that also have a second therapeutic action. For instance, 5-FU has been loaded in iron oxide nanoparticles, able to produce magnetic hyperthermia that effectively reduces tumor growth in heterotopic human colon cancer mouse models [87]. Also, poly (lactic-co-glycolic acid) (PLGA) has been approved by the United States FDA for oral drug delivery; it has EGF-functionalized and coloaded with 5-FU and perfluorocarbons to inhibit colon tumor growth [88]. Dendrimers, highly branched spherical molecules, have been largely employed in the active targeting of different drugs to CRC cells [89,90,91].

Finally, the above discussed mesoporous silicas offer remarkable advantages and biosafety in CRC treatment and their potential clinical application value is high [92]. 

CRC, due to its character of a heterogeneous disease, presents complex needs in which multiple-level molecular mechanisms play a role and should be considered to plan an efficient, in principle resistance-free, therapeutic approach. The recently introduced solutions of nanotechnology-assisted therapeutics concern the engineering of chemotherapeutics release. 

### 7.2. Comparison Between Nanotechnology-Based Therapies and Conventional CRC Treatments

One of the most common advantages of the use of nanomedicine is a reduction in the drug dose quantity. In conventional therapy with 5-FU, the dose is much higher and more toxic compared to nanoencapsulation of 5-FU [93]. Furthermore, nanotechnology-based therapies allow enhanced solubility and stability and selective delivery to target tissues, avoiding healthy tissues, which results in an improved pharmacokinetic profile, increased drug bioavailability, reduced drug dose, enhanced efficacy and reduction of adverse effects [94]. On the other side, nanoparticles should not accumulate in the organism, so biodegradable materials should be preferred. In our experience [95], the development of nanodevices starting from mesoporous silica nanoparticles with amorphous defective frameworks has not provided evidence of silicon detection in the treated tissues. This is due to the fact that, different from crystalline silicate such as zeolites [96], the amorphous mesoporous silicas are degradable by hydrolysis in a particular way during the synthesis procedure, which, in addition to its simplicity, has shown high reproducibility and scalability, favoring the formation of a defective framework. Nevertheless, the application of nanomedicine solutions to large-scale industrial production remains challenging due to the need for an important adaptation of regulatory guidelines to nanomaterial-based drugs [97].

This perspective is based on the significant advantages of nanotechnology instruments: the sustainable potentiality of multidrug release through mesoporous silicas that are able to carry different drugs, diffuse, and reach the tissues of therapeutic relevance without toxic effects [95].

### 7.3. Nanotechnology Application in Early Colorectal Cancer Detection

CRC is a disease where early detection and prevention play a critical role in improving outcomes. In many cases, the cancer is diagnosed only after lymph node or distant metastasis has occurred, making treatments less effective [98]. Detection of the cancer in the early stages significantly reduces the CRC mortality rate, improves patients’ quality of life and also provides a better chance of early treatment [99].

Various screening methods have been established to detect polyps and adenomas at early stages, allowing for their removal before the development of CRC. Primary screening techniques include stool testing, flexible sigmoidoscopy, colonoscopy, computed tomography, and double-contrast barium enema. DNA markers research, such as ITG4 methylation, SFRP2 methylation, miR-21, miR-92a, miR-135b, and Cologuard, can help in detection of the disease [100]. 

The CellSearch® test is based on circulating tumor cells (CTCs) and provides predictive information for metastatic CRC. Currently, various systems including MagSweeper, Cynvenio, IsoFlux, VerIFAST, Adnagen, and magnetic sifters have been developed to improve the detection speed and efficiency [101]. Each method offers distinct advantages and challenges, influencing its suitability for different patients and clinical scenarios. Therefore, it is essential to develop screening tests for CRC that are more sensitive, rapid, inexpensive, and specific.

Diagnostic imaging is the area where nanotechnologies have the potential to make their most impactful contribution. Nanotechnology interventions in CRC play a key role in tumor screening using nanomaterials, providing suitable tools with improved sensitivity, lower toxicity, enhanced permeability, better tissue penetration and more precise targeting in tissues [102,103].

Today, various types of organic and inorganic nanoparticles are available for CRC diagnostic purposes, featuring a wide range of sizes, structures and compositions.

Yamashota and coworkers developed fluorescent nanospheres suitable as imaging agents for fluorescence colonoscopy in the detection of early colorectal cancer. The agent is composed of submicron-sized fluorescent polystyrene nanospheres with two functional groups, peanut (Arachis hypogaea) agglutinin (PNA) and poly(N-vinylacetamide) (PNVA) on their surfaces. PNA acts as a targeting moiety that specifically binds to the Thomsen–Friedenreich (TF) antigen expressed on the mucosal side of colorectal cancer cells through the agent’s recognition of Gal-β(1–3)GalNAc, the terminal sugar of the antigen, while PNVA enhances the specificity of PNA by reducing the nonspecific interaction between the imaging agents and normal tissues [104]. Novel in vitro studies on the interactions between imaging agents and various cultured human cell types demonstrated the specificity of the imaging agent in strongly binding to cancer cells that express the TF antigen, Furthermore, in vivo studies revealed that the imaging agent selectively and with high affinity bound to HT-29-RFP-derived tumors implanted orthotopically in the large intestine of nude mice [105].

Surface-enhanced Raman spectroscopy (SERS) greatly improves the detection sensitivity of Raman spectroscopy and has drawn considerable attention due to its great potential in biomedicine. Duo Lin et al. conducted an exploratory study, pioneering the development of a gold nanoparticle-based SERS serum analysis combined with principal components analysis (PCA) and linear discriminant analysis (LDA) diagnostic algorithms. The method was able to differentiate colorectal cancer from normal tissue with high diagnostic sensitivity and specificity. This approach aims to create a clinical tool for non-invasive detection and screening of colorectal cancers [106].

Chen et al. developed novel polyp-targeting, fluorescently-labeled mesoporous silica nanoparticles (MSNs) to serve as targeted endoscopic contrast agents for the early detection of polyps and nascent colorectal cancer. FITC was first co-condensed into the silica framework of MSNs to enable their fluorescence tracking both in and ex vivo, while MSNs were then coated with two different lengths of polyethylene glycol (PEG) polymers to increase their water solubility, aiding their diffusion through mucus. Lastly, fluorescent/PEGylated MSNs were labeled with Ulex Europaeus Agglutinin-1 (UEA1) for targeting premalignant lesions. In vitro cell studies, ex vivo histopathological analysis, and in vivo colonoscopy and endoscopy of murine colorectal cancer models demonstrated significant binding specificity of the nanoconstruct to pathological lesions via targeting aberrant α-L-fucose expression [107].

Early-stage CRC can be diagnosed through in vitro cellular assays, in vivo solid tumor MRI, and ex vivo tissue biopsy analysis by using a unique nanoprobe synthesized by Cheng and his team. The molecular nanoprobe, GdDTPA∙BSA@QDs-PcAb, consists of a fluorescent quantum dot (QD) core, a coating layer of paramagnetic DTPA-Gd coupled BSA (GdDTPA∙BSA), and a surface targeting moiety of anti-Glut1 polyclonal antibody. In in vivo MRI studies, the potential of GdDTPA∙BSA@QDs-PcAb as a promising candidate for CRC contrast-enhanced MRI diagnosis has been demonstrated. Additionally, it can be employed for analyzing tumor biopsy tissue specimens [108].

A study by Pan developed a simple and highly sensitive method for detecting aldo-ketoreductase family 1 member B10 (AKR1B10) in serum, which is a prognostic marker and therapeutic target for colorectal cancer (CRC). The method utilizes quantum dots (QDs) with a high fluorescence quantum yield that resists photo-bleaching and provides size-controlled luminescence. This immunofluorescence assay, which uses anti-AKR1B10-conjugated CdTe/CdS QDs, showed promise for the early detection of CRC. The technique is fast, easy to implement, and offers high sensitivity and specificity [109].

Lectin conjugated on Fe_2_O_3_@ nanoparticles (lectin–Fe_2_O_3_@Au NP) were synthesized and used as molecular probes for in vivo magnetic resonance (MR) and computed tomography (CT) dual imaging. Systematic studies demonstrated that lectin–Fe_2_O_3_@Au NPs exhibit excellent biocompatibility, good contrast agent property, and long-term colloidal stability in different media, including water, PBS, and culture medium, demonstrating promising for both in vitro and in vivo MR and CT imaging of CRC [110].

Margel presents the development of new NIR fluorescent proteinoid-poly(L-lactic acid) (PLLA) nanoparticles. A P(EF-PLLA) random copolymer was synthesized by thermally copolymerizing L-glutamic acid, L-phenylalanine, and PLLA, forming self-assembled nanosized hollow particles. These nanoparticles encapsulated the NIR dye indocyanine green, significantly enhancing its photostability. The nanoparticles were stable, non-toxic, and showed no dye leakage in phosphate-buffered saline with human serum albumin. Tumor-targeting ligands, including peanut agglutinin and anti-carcinoembryonic antigen antibodies, were conjugated to the nanoparticle surface, improving tumor fluorescence. Specific colon tumor detection was successfully demonstrated in a chicken embryo model [111].

### 7.4. Possible Synergetic Actions to Overcome Multiple Level Resistance Mechanisms

Single-agent KRAS G12C inhibitors (sotorasib and adagrasib) have shown improved outcomes in patients with non–small-cell lung cancer with KRAS G12C mutation but limited activity in CRC patients. 

Sotorasib plus panitumumab, according to an approach of dual KRAS G12C and EGFR blockade, overcame treatment resistance in patients with colorectal cancer with KRAS G12C mutation, typically a population not responding to EGFR inhibitors, such as cetuximab and panitumumab [112,113].

RAS mutated cancers do not respond to anti-EGFR drugs. The mutation in the RAS oncogene, revealed in more than 50% of mCRC cases, is still the unique validated biomarker of resistance to anti-EGFR monoclonal antibodies (mAbs). Circulating tumor DNA (ctDNA) studies, in cases of acquired resistance to anti-EGFR therapies, have allowed the identification of genomic alterations such as RAS and other molecular drivers, in tumors initially diagnosed as wild type (WT) [114]. The detection of RAS-mutated clones associated with the presence of EGFR extracellular domain (ECD) mutations that impede the binding of mAbs to the EGFR receptor in approximately one-third of cases represents another resistance mechanism [115].

BRAFV600E mutations, occurring in 8–10% of mCRC and frequently associated with MSI and RAS WT tumors, determine a worse prognosis and could predict a poorer response to anti-EGFR treatment [116,117].

**Figure 7:**
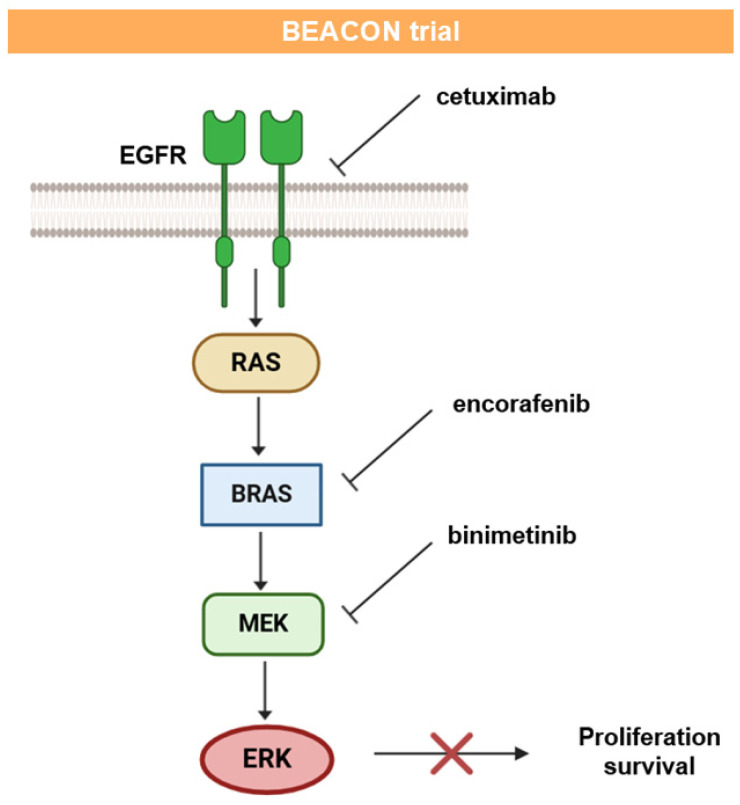
Triple combination therapy involving anti-EGFR, BRAF, and MEK inhibitors for patients with BRAF V600E-mutant metastatic colorectal cancer in the BEACON trial.

The BEACON trial evaluated encorafenib and cetuximab +/− binimetinib (triplet or doublet combination). Its rationale is schematically presented in Figure 7.

The triple combination of encorafenib, binimetinib, and cetuximab confirmed a 27% ORR and the encorafenib plus cetuximab doublet had a 20% ORR, with an acceptable toxicity profile [118,119]. Double and triple combinations showed prolonged maintenance of quality of life (QoL) [120]. The patients with BRAF V600E CRC could benefit from a triple combination of targeted therapy, avoiding the need for chemotherapy and its toxic side effects, changing the course of this very aggressive disease.

Combination therapies among chemotherapies and targeted therapy were introduced several times. They generally show improvements in patient outcomes. On the other side, in the biological therapy’s world, as in the case of the BEACON trial, it is well-known that combined therapies univocally show advantages over monotherapies [121]. In this perspective paper, the complete recovery with fully biological instruments by means of a multidrug inhibiting the initiation, progression, and migration of CRC is hopefully planned in the future.

Next-generation sequencing (NGS) technology allows the simultaneous identification of multiple genetic mutations and fusions involved in the oncogenesis of different neoplasms. It provides complete information on tumor biology, guiding the choice of treatment with a single analysis. Despite a limited number of molecular targets in CRC, NGS technology remains the recommended approach to optimize workflows in different malignancies. NGS technology can detect rare or unusual mutations that may be missed by other screening methods. It improves diagnostic and therapeutic choices, expanding therapeutic options, including those that are newly approved. Due to the fact that cancer progression is the result of a series of consecutive events represented by mutations, its dynamic character is the real enemy to be fought by maintaining reasonable expectations of avoiding chemoresistance. The monitoring of molecular signatures is the basic instrument for the development of personalized drugs. Thus, biomarkers become the main elements for drug development and patient stratification, revealing the new cancer identity to be considered.

Furthermore, a completely new approach to enrolling patients in clinical trials is needed. 

The solution for this need is a multi-drug, as multiple drivers define cancer evolution. We believe that, based on the limited action potentialities of drug molecules in living organisms, especially when they are large molecules, chimeric ones or multi-drugs, and with the recent advances of nanotechnology, an efficient multi-drug administration cannot ignore the possibility of developing a Multitarget Drug Delivery Device. 

The authors glimpse a triple synergistic potential in future development of multitargeting agents for CRC and other cancer-related Multiple Level Resistance Mechanisms. This synergistic action lies between the activity of small molecules and antibody-mimicking, peptides, and the tumor-mapping potentialities of NGS, and, on the other side, the drug release ability of nanostructured vectors such as engineered mesoporous silica that would allow the release of multiple drugs in the TME [121,122].

**Figure 8:**
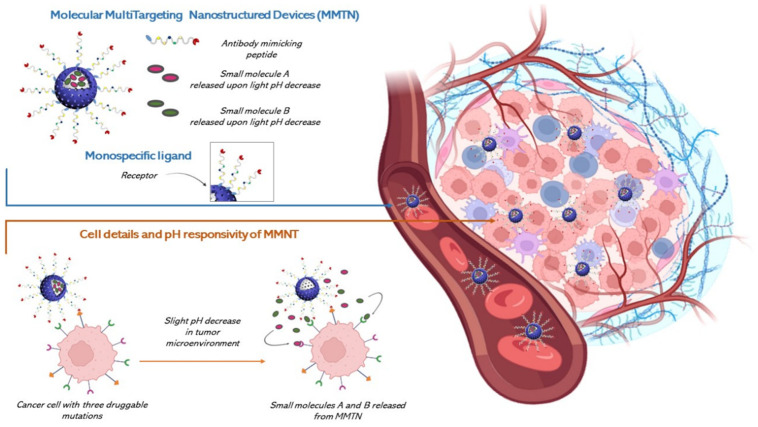
Graphical representation of the imaginary molecular multi-targeting nanodevice (MMTN) envisioned as a potential future advancement in the field of molecular targeted therapy.

A common need has arisen from the discussion of the main critical druggable pathways and the occurrence of drug resistance. Although our discussion is not comprehensive, we believe that the future CRC therapeutic direction is multiple level targeting, whose urgent need is a multi drug approach. Theranostics and therapeutics for cancer in general and CRC in specific could significantly benefit from nanotechnology, as it would enable more targeted, individualized care with fewer side effects [85,123]. The chemical biologist is used to find a solution in the world of chimeric or multidrugs while a material chemist would suggest a nanovehicle able to reach the TME and there release these drugs. We have already presented this imaginary solution [121]; it is based on a series of well-verified nanotechnological elements and solutions, joined and engineered through the bottom-up approach, and this makes it the nearest to the real world. Here again we present a new opportunity for cancer therapeutics based on a Molecular Multi Targeting Nanostructured Device (MMTN), an efficient vehicle for reaching the TME and there release a multi drug payload that, in the case of CRC, is a very urgent need. The starting nanoarchitecture is a biocompatible and biodegradable MSU-x type mesoporous silica obtained through a particular biphasic synthesis at the interface, using Triton X-100 (TX-100) a nonionic surfactant at room temperature [124].Figure 8 shows the solution we announced [121].

## 8. Conclusions

In this feature paper, there arises a strong indication toward somatic and germline pancancer multigene panel testing supported by specialized genetic counseling services for patients diagnosed with early onset colorectal cancer. Due to the increasing level of knowledge, the potentialities of identification of a germline pathogenic variant in a known hereditary cancer gene should drive the clinical management of all patients and their relatives. The targets involved, in fact, that are related to somatic mutations, significantly contribute to the definition of the prognosis and should determine the surgical and other therapeutic decisions. 

Furthermore, it is very likely that a deeper understanding of the various molecular subtypes of CRC may require multi-drug therapies in the future, enabling the benefit of synergistic effects between drugs and nanotechnological tools that allow for very fine materials engineering [91] that is certainly capable of satisfying the most complex needs of multidrug therapies. We believe it is possible to translate nanotechnological know-how from the world of nanomedicine to the world of molecularly targeted therapies for CRC and other types of tumors [119].
